# Turning by External Manipulation

**Published:** 1850-10

**Authors:** 


### OBSTETRICS.

*Turning by External Manipulation*—The endeavor to rectify the faulty position of the fœtus in utero, by external manipulation, engaged much of the attention of the older practitioners, and especially of Wigand in 1807, but is scarcely alluded to in modern works on midwifery. Professor Martin, of Jena, has recently published an interesting essay upon the subject, based upon twenty-seven cases related by other authors, and seven which have occurred to himself. In these all the mothers did well, as also all the children but four, of which number, too, one was already dead, and another was delivered by perforation.

It is evident that an exact knowledge of the position of the child must be obtained before the attempt is made, and Professor Martin be lieves it is preferably acquired by external examination, although this implies a certain amount of dexterity. It is only prior to, or immediately after, the discharge of the waters, that the fœtus will be found possessed of sufficient mobility. As long as the os is undilated, and the pains irregular, the patient is kept on the side upon which the part desired to be forced into the pelvis is placed, so as to afford a moderate support. When, however, the os has become dilated, and the waters are expected to be discharged, she is laid (the bladder and rectum having been emptied) on her back the lower part of her body being somewhat raised. With one hand the operator presses the part of the fœtus which lies nearest the os, whether it be the head or breech, downwards,

61*

while, with the other, he presses the rest of the body upwards. This simultaneous pressure is begun during the interval of a pain, and continued during its commencement, while, during the height of this, the uterus is firmly supported on every side. After a short pause the manipulation is again recommenced, and, if the operator's hands become tired, an assistant may, during the intervals of the pains, support the belly on each side, whereby the ovoid configuration of the uterus is better secured, and the child more easily brought into a proper position. If the pains are long absent, she may often be advantageously placed on her side, supporting the projecting part by the hand or a firm cushion. Especially is this position advantageous if manipulation has already somewhat improved the position, and it should, if possible, be continued after the head has entered the pelvis. For the purpose of retaining the head within the pelvis, when the improvement of the position is not durable, the rupture of the membranes, after the head or buttocks have been brought over the os, is a very efficacious procedure.

Among the rules to be observed, there is that of never employing a cold hand, for, not only is it disagreeable, but it may give rise to spasmodic action and a premature discharge of the waters. The pressure, too, should be moderate and continuous, and applied sometimes in a diffused manner with the flat hand, and at others by particular fingers, to various parts of the child. It must be always a double and simultaneous pressure, exerted equally towards the fundus and upon the part we are seeking to engage in the pelvis. A change in the position of the woman is often an important aid to the manipulation.

There are certain conditions requisite for rendering the operation of external turning an eligible one.—1. Absence of reasons for a rapid termination of labour, as hæmorrhage, presentation of funis. 2. Mobility of the child. Generally this ceases after a discharge of the waters. If, however, few pains have occurred, the presenting part has not become far forced down, or the uterus firmly contracted around it, a trial is justifiable. 3. Absence of great sensibility of the womb or abdomen. 4. Sufficient capacity of pelvis. A moderate degree of contraction is no contra-indication. 5. A normal activity of pains has been set down as a condition. It certainly is a very favorable one, but it is rare in faulty presentation, and not an essential one for external turning. Spasmodic or crampy pains which render the first two stages tedious, if due to cold, are best treated by mustard poultices and small doses of ipecacuanha. The very rectification of the position sometimes imparts a normal activity to the pains. External turning is rarely successful when the defective pains are dependent upon a rheumatico-catarrhal affection of the lower portion of the uterus giving rise to a softened state and premature rupture of the membranes. 6. The child being alive is a subordinate condition; for, although in the case of its death, internal turning may usually be advised, yet we very frequently cannot be certain of this.—*Dublin Med. Times, from Froriep's Notizien.*

